# Determination of L-Argininamide Based on Water-Soluble Fluorescent Conjugated Polymer-Aptamer

**DOI:** 10.1155/2013/682134

**Published:** 2013-08-20

**Authors:** Hongliang Guan, Zhike He

**Affiliations:** ^1^School of Environment and Civil Engineering, Wuhan Institute of Technology, Wuhan 430074, China; ^2^Key Laboratory of Analytical Chemistry for Biology and Medicine (Ministry of Education), College of Chemistry and Molecular Sciences, Wuhan University, Wuhan 430072, China

## Abstract

Water-soluble fluorescent conjugated polymer is a promising material which could be used as an optical platform in highly sensitive molecular sensors. In this paper, a simple label-free DNA sensor, which consisted of a poly(3-alkoxy-4-methylthiophene) and an aptamer, was used to detect L-argininamide (L-Arm). Due to the specific binding reaction between L-Arm and its aptamer, the proposed method can easily determinate the L-Arm through the recovery of fluorescence without any modification. Other ions or similar molecules had little effect on the detection. Moreover, there was a linear relationship between fluorescence intensity and the concentration of L-Arm. The detection limit of L-Arm was as low as 4.7 nM.

## 1. Introduction

Recently, the use of water soluble fluorescent conjugated polymers (CPs) as either chemical or biological sensing elements has received wide interest [1–5]. CPs contain a large number of absorbing and delocalized molecular units, and the transfer of excitation energy along the whole backbone of the CP to the energy/electron acceptor results in an amplification of fluorescence signals. Therefore, CPs have been successfully employed in the detection of various substances including DNA, RNA, protein, metal ions, and even pH and temperature [6–9].

Aptamer, a kind of one single-stranded DNA or RNA sequences, can be synthesized with the systematic evolution of ligands by the exponential enrichment (SELEX) procedure [10, 11]. The unique properties of aptamer is that it can bind with a variety of targets ranging from small molecules, proteins, and even to cells at very high affinity. Unlike ELISA, immunobead assay, and western blotting, some unspecific binding will occur in such cases, aptamers provide decisive advantages. First, they are more resistant against denaturation and degradation. Second, their binding affinities and specificities can be manipulated easily and improved by rational design or by techniques of molecular evolution. Therefore, aptamer is widely utilized as recognition molecular for detecting a variety of targets ranging from small molecules, proteins, and even cells [12–17]. 

In this paper, we propose utilizing water-soluble fluorescent polythiophene derivative and aptamer for detecting L-Arm. On one hand, thiophene polymer can be easily prepared through oxidation of the corresponding monomers; on the other hand, thiophene polymer can detect, transduce, and possibly amplify chemical, biological, and physical changes into measurable optical or electrical signals with very high sensitivity [18–21].

## 2. Materials and Methods

Poly(1H-imidazolium-1-methyl-3-{2-[(4-methyl-3-thienyl)-oxy]ethyl})chloride (PT) was synthesized according to the previously published literatures [18]. The stock solution of PT (2 × 10^−4^ M, per repeat unit) was dissolved in pure water and used by appropriate dilution, all the oligonucleotides (aptamer, A1, A2, A3, and A4, listed in Table 1. Of the four aptamers, A3 was an unrelated one that was designed for control experiment) solutions were prepared in 10 mM Tris-HCl (pH 7.4, containing 0.01 M NaCl) buffer solution. For fluorescence assay, in 5 mL eppendorf tube, a 20 *μ*L of PT solution was added to 4 mL Tris-HCl aqueous buffer solution, followed by the addition of 10 *μ*L aptamer (A1–A4) and L-Arm, respectively. The final concentration of PT was 1.0 × 10^−6^ M, L-Arm was 1.0 × 10^−6^ M, and aptamer was 3.3 × 10^−8^ M. The mixtures were incubated in water at 45°C for reaction and detection.

L-Arm and all oligonucleotides were purchased from a biological engineering cooperation. The concentration of DNA was determined by measuring the absorbance at 260 nm in a 3 mL quartz cuvette. Tris(hydroxymethyl)aminomethane (Tris) was purchased from a chemical company. Unless other specified, the rest reagents were analytical grade and used without further purification. The water used was purified through a purification system (≥18 MΩ). Fluorescence measurements were acquired in 3-mL quartz using a luminescence spectrometer equipped with a temperature-controlled cuvette holder and a circulating bath. The pH of solution was measured with a pH meter.

## 3. Results and Discussion

**Figure 1:**
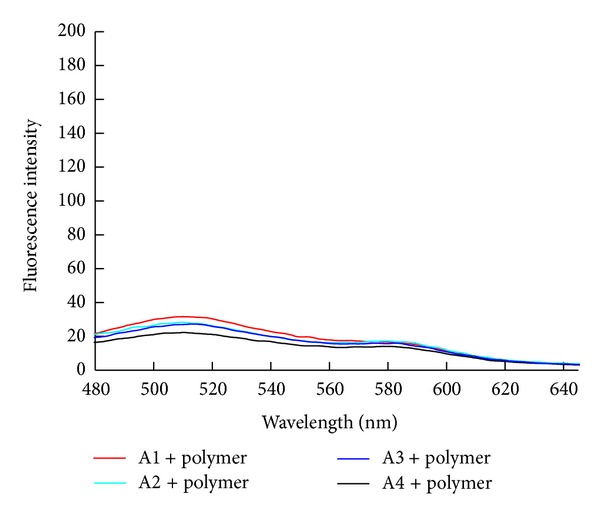
The fluorescence spectra of thiophene polymer in a different aptamer solution. The concentrations of polymer and aptamer were 1.0 × 10^−6^ M and 3.3 × 10^−8^ M. Tris-HCl was 0.01 M and contained 0.1 M NaCl, pH 7.4 at room temperature.

**Figure 2:**
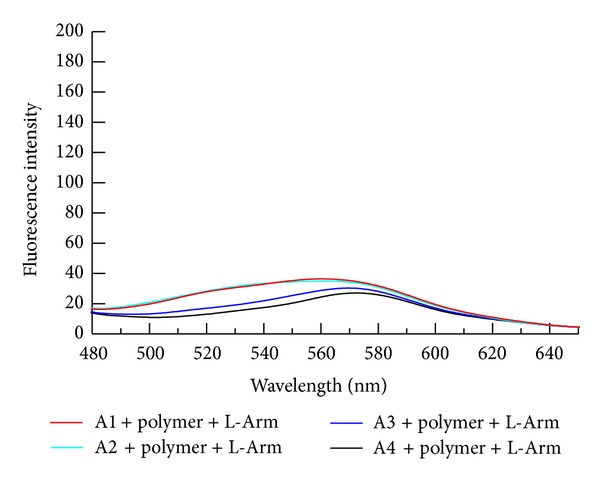
The fluorescence spectra of thiophene polymer in different aptamer and L-Arm solutions. The concentrations of polymer, L-Arm, and aptamer were 1.0 × 10^−6 ^M, 1.0 × 10^−6 ^M, and 3.3 × 10^−8 ^M, respectively. Tris-HCl was 0.01 M and contained 0.1 M NaCl, pH 7.4 at room temperature.

**Figure 3:**
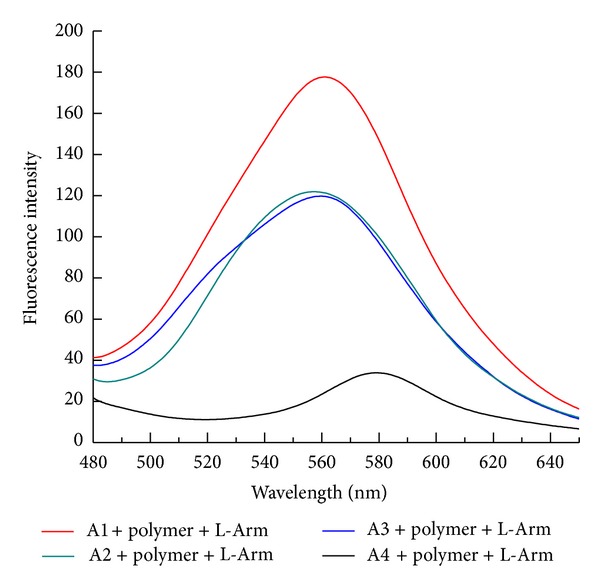
The fluorescence spectra of thiophene polymer in a different aptamer solution. The concentrations of polymer, L-Arm, and aptamer were 1.0 × 10^−6^ M, 1.0 × 10^−6^ M, and 3.3 × 10^−8^ M, respectively. Tris-HCl was 0.01 M and contained 0.1 M NaCl, pH 7.4. Detection temperature was 45°C.

As shown in Figure 1, at room temperature, mainly due to the electrostatic attraction between positively charged PT and negatively charged aptamer, the fluorescence of PT in each solution was weak, even in the presence of L-Arm (Figure 2). when the solution was heated to 45°C, the fluorescence of PT was recovered to a certain extent. As shown in Figure 3, the fluorescence intensity of PT in the solution containing the hairpin conformation aptamer A1, PT, and L-Arm was the highest, followed by A2 and A3, and the fluorescence of solution containing the linear conformation aptamer A4 showed no pronounced change. Therefore, A1 was chosen as the best candidate for the detection of L-Arm. Besides the different fluorescence intensity, an evident red shift was also observed. Unlike detection of mismatched or perfectly matched ssDNA in the published literature [23], in which the maximum emission is restored to be at 520 nm, in this experiment, the maximum emission of PT was located at 560 nm.

### 3.1. The Effect of Temperature on the Fluorescence

**Figure 4:**
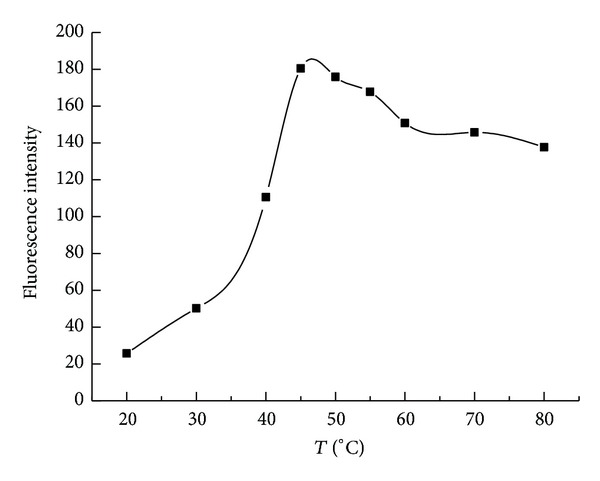
The influence of temperature on the fluorescence intensity. The concentrations of polymer, L-Arm, and A1 were 1.0 × 10^−6 ^M, 1.0 × 10^−6 ^M, and 3.3 × 10^−8 ^M, respectively. Tris-HCl was 0.01 M and contained 0.1 M NaCl, pH 7.4. Detection temperature was 45°C.

Only under a certain temperature, PT, L-Arm and A1 could constitute a stable complex, and the fluorescence of the system recovered to maximum level. Therefore, proper reaction temperature was important for fluorescence recovery. As shown in Figure 4, with the increase of temperature, the fluorescence intensity of the system enhanced gradually, especially in the range of 45°C–50°C, and the fluorescence intensity reached the maximum. However, when the temperature rose higher, the fluorescence intensity of the system decreased a little. One possible reason was that the structure of the system might be destroyed under the higher temperature. Therefore, the temperature of 45°C was chosen for the experiment.

### 3.2. The Influence of pH

**Figure 5:**
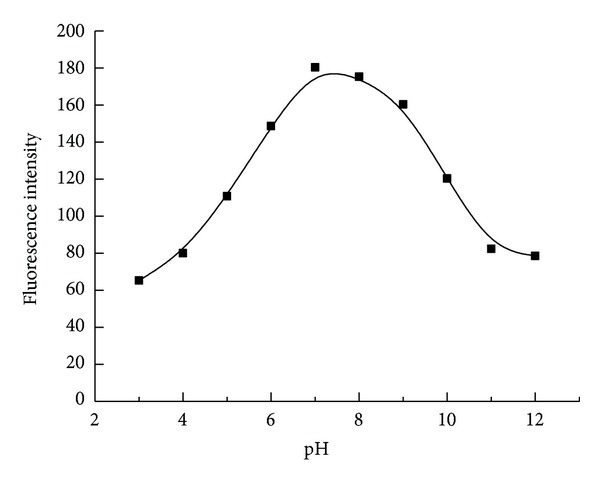
The influence of buffer on the fluorescence intensity. The concentration of polymer, L-Arm and A1 were 1.0 × 10^−6 ^M, 1.0 × 10^−6 ^M and 3.3 × 10^−8 ^M, respectively. Tris-HCl was 0.01 M contained 0.1 M NaCl. Detection temperature was 45°C.

pH of the buffer system was another factor which could have potential impact on the intensity of fluorescence. In order to investigate the influence on recovery efficiency, a different pH value of the buffer solution was prepared for the experiment.  Figure 5 indicated that the neutral pH was helpful for the recovery of the fluorescence. In the following experiments, the buffer of pH 7.4 was used for the detection.

### 3.3. The Mechanism Discussion

**Figure 6:**
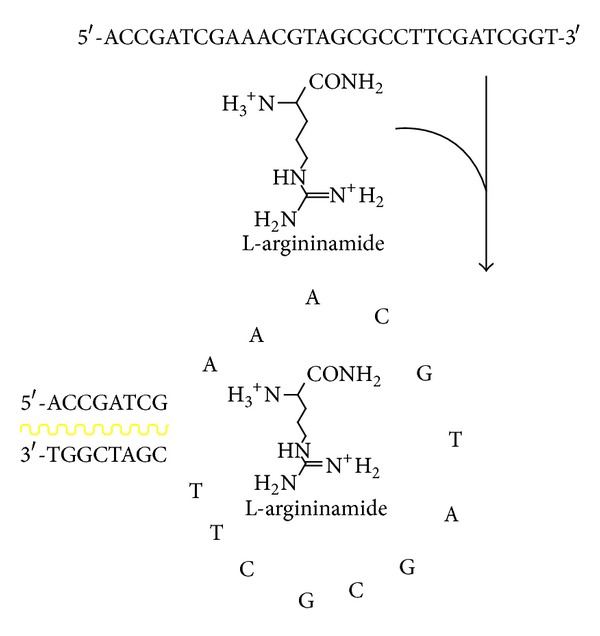
Schematic description of the interaction between aptamer, polymer and L-Arm.

Under room temperature, even with the addition of L-Arm to the solution consisting of A1 and polymer, the fluorescence of the system could not be recovered. Only under a certain circumstance, for example, after heating, A1, polymer, and L-Arm developed a loop formation (Figure 6), and the bases matched with each other to form a hydrophobic region and a closer structure, and the fluorescence of the system could be recovered. Moreover, L-Arm played a vital role for stabilizing the structure via hydrogen bond with the right aptamer. The aptamer with loop, or other aptamer without loop, even under the previously mentioned conditions, could not form a more stable structure, and thus the fluorescence of the system would not recover completely.

### 3.4. Linear Relationship and Detection Range

**Figure 7:**
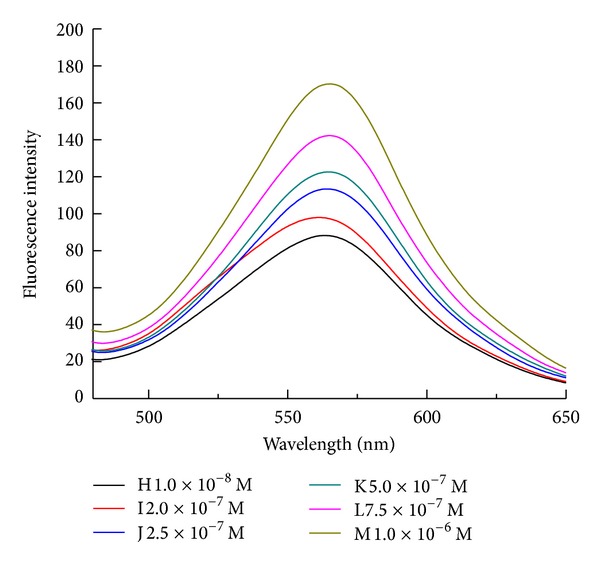
Fluorescence spectra of the system after the addition of different concentration of L-Arm.

**Figure 8:**
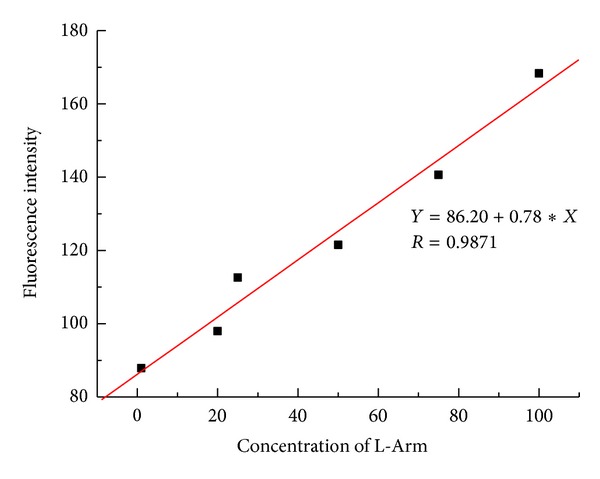
The linear plots of increased fluorescence intensity at the different concentration of L-Arm.

Since the addition of L-Arm could make the fluorescence recovery, it was hoped that the aptamer and polymer composite could quantitatively reflect the amount of L-Arm added. As shown in Figures 7 and 8, by monitoring the fluorescence intensity at 514 nm, a good linear relationship was obtained in the range from 1.0 × 10^−8^ M to 1.0 × 10^−6^ M. The limit of detection at an S/N ratio of 3 was 4.7 nM.

### 3.5. Specific Investigation

**Figure 9:**
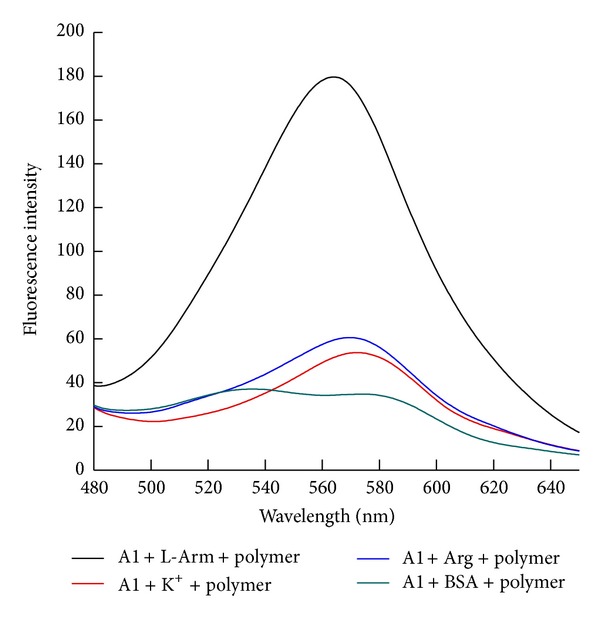
The effect of different disturbances on the inflorescence of the system.

In order to verify the specific detection of the aptamer to L-Arm, three controlled experiments with addition of K^+^, arginine, and BSA (bovine serum albumin) were carried out under identical conditions. In the previous studies, single-stranded DNA with G-rich sequences was able to fold into secondary structures quadruplex via intramolecular hydrogen-bonding interactions in the presence of K^+^ [24]. When the potassium ion specifically bound to the G-quadruplex DNA, the fluorescence of the system was recovered to the high point by using cationic polyfluorene. In this experiment, the binding sequence A1 also contained plenty of guanine. Arginine, an amino, whose structure was very similar to that of L-Arm and BSA, a commonly used protein in the lab, was also chosen for deciding the binding specificity. In these cases (seen in Figure 9), the addition of K^+^, arginine, and BSA could not render the fluorescence recover, and only in the presence of L-Arm, the fluorescence of the system could recover to the maximum, which indicated that the polymer and aptamer system could offer a specific approach for detection of L-Arm.

The concentrations of polymer, L-Arm, A1, K^+^, Arg, and BSA were 1.0 × 10^−6^ M, 1.0 × 10^−6^ M, 3.3 × 10^−8^ M, 1.0 × 10^−6^ M, 1.0 × 10^−6^ M, and 1.0 × 10^−6^ M, respectively. Tris-HCl was 0.01 M and contained 0.1 M NaCl, pH 7.4. Detection temperature was 45°C.

## 4. Conclusion

In summary, we have developed an exceptionally simple, rapid, and specific method for detection of L-Arm without any complicated protocols or laborious modification. When aptamer, a special oligonucleotide, quenched the fluorescence of the polymer, the addition of L-arm could make the fluorescence recover under a certain condition. The quenching and recovery mechanisms are ascribed to the electrostatic interactions and energy transfer between polymer and aptamer. Moreover, the recovered emission intensity is proportion to the concentration of L-Arm, whereas a new homogeneous assay measurement for L-Arm is established with a LOD as low as 4.7 nM. Since other substances did not have the obvious influence on the fluorescence of the system, the method could be applied for specific detection of L-Arm.

## Figures and Tables

**Table 1:** Aptamer sequences used in the experiment from the literature [22].

Name	Sequence	Conformation
A1	ACCGATCGAAACGTAGCGCCTTCGATCGGT	Hairpin
A2	AGCGATCGAACGTCACCGGATTCGATCGCT	Hairpin
A3	AGACCAGGGCAAACGGTAGGTGAGTGGTCT	Hairpin
A4	GATCGAAACGTAGTCTCCCGATCGCATCGT	Linear
